# *µ*-XRF Studies on the Colour Brilliance in Ancient Wool Carpets

**DOI:** 10.1155/2017/6346212

**Published:** 2017-01-10

**Authors:** Markus Meyer, Camelia N. Borca, Thomas Huthwelker, Manfred Bieber, Karl Meßlinger, Rainer H. Fink, Andreas Späth

**Affiliations:** ^1^Physikalische Chemie II and ICMM, Friedrich-Alexander-Universität Erlangen-Nürnberg (FAU), Egerlandstraße 3, 91058 Erlangen, Germany; ^2^Swiss Light Source (SLS), Paul Scherrer Institute, 5232 Villigen, Switzerland; ^3^Ex Oriente, Waldleite 17, 97295 Waldbrunn, Germany; ^4^Physiologie und Pathophysiologie, Friedrich-Alexander-Universität Erlangen-Nürnberg (FAU), Universitätsstraße 17, 91054 Erlangen, Germany; ^5^CENEM, Friedrich-Alexander-Universität Erlangen-Nürnberg (FAU), Egerlandstraße 3, 91058 Erlangen, Germany

## Abstract

Many handmade ancient and recent oriental wool carpets show outstanding brilliance and persistence of colour that is not achieved by common industrial dyeing procedures. Anthropologists have suggested the influence of wool fermentation prior to dyeing as key technique to achieve the high dyeing quality. By means of *μ*-XRF elemental mapping of mordant metals we corroborate this view and show a deep and homogenous penetration of colourants into fermented wool fibres. Furthermore we are able to apply this technique and prove that the fermentation process for ancient specimens cannot be investigated by standard methods due to the lack of intact cuticle layers. This finding suggests a broad range of further investigations that will contribute to a deeper understanding of the development of traditional dyeing techniques. Spectroscopic studies add information on the oxidation states of the metal ions within the respective mordant-dye-complexes and suggest a partial charge transfer as basis for a significant colour change when Fe mordants are used.

## 1. Introduction

**Figure 1:**
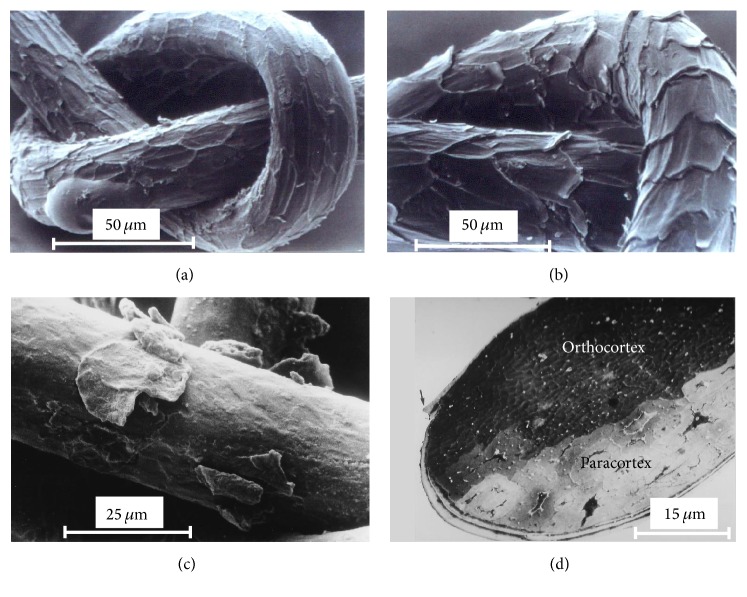
(a) Scanning electron micrograph of Anatolian sheep wool fibres recently dyed in a bath of madder roots and KAl(SO_4_)_2_. No fermentation process; the cuticle scales are closely attached to the fibre. (b) 20 days of fermentation with* G. candidum* prior to dyeing; abduction of cuticle scales from the fibre. (c) Carpet fibre from 18th century; cuticle scales mainly lost due to mechanic abrasion. (d) TEM micrograph of the cross-section of a sheep wool fibre depicting cuticle layers and cortex. The visualization of ortho- and paracortex is enhanced by contrast agents (lead citrate and uranyl acetate). OsO_4_ was applied for fixation.

Not only the elaborate patterns, but also the vivid and persisting colours of oriental carpets and other flat weaves have been fascinating people all over the world since medieval times. Many of these textiles are remarkably stable against any typical forms of bleaching, either photon-induced or leaching, and keep their shiny colour brilliance over centuries even under harsh conditions. This observation pertains especially to preindustrial flat weaves that have been manufactured based on ancient dyeing techniques. Since the 1970s cultural anthropologists have studied and revived traditional Anatolian dyeing procedures in the Kavacık project [1]. They collected and evaluated methods for dyeing of hand-prepared sheep wool with locally available natural dyes and found that the key technology for the persistent colour brilliance of the investigated textiles was a controlled fermentation of the wool with* Geotrichum candidum* yeast prior to dyeing [2]. Scanning electron microscopy (SEM) revealed that the fermentation procedure leads to decomposition of fatty interstices in between the cuticle scales of the wool fibres resulting in scales abduction and higher permeability of the cuticle [1, 3]. Furthermore, diffusion of cationic species into hair fibres takes place mainly in the nonkeratinous components of the fibres [4] and is hindered by a high concentration of cysteic acid in the cuticle (especially in weathered fibres) [5, 6] that binds metal ions at moderate and high pH [7]. Therefore, diffusion of such species deep into the fibre cortex can be fostered by a relatively low pH as it is induced by the yeast culture [1]. The increased permeability of fermented wool could be shown by diffusion experiments subsequently analysed by means of transmission electron microscopy of cross-sectioned wool fibres [8]. Due to this enhanced permeability, the colourants can protrude to interstices and the cell membrane complex deep inside the cortex of the hair fibres during the following dyeing process and an almost homogeneous distribution of the dye along the fibre diameter is achieved. The characteristic change of the wool cuticle surface is a clear indicator for previous fermentation and helps to verify the manufacturing procedure of a textile specimen (cf. Figures 1(a) and 1(b)). However, the cuticle layer of wool fibres in long-term used textiles is often lost by abrasion and cannot be investigated any more (cf.  Figure 1(c)). Furthermore, the proof of enhanced diffusion by TEM works just for controlled conditions. In the case the treatment of the specimen is not known, the chemical nature of the penetrated material has to be revealed. We addressed these challenges by a direct detection of colourants in the inner hair matrix with high chemical sensitivity.

Within the present study we investigated Anatolian sheep wool fibres that had been dyed with extracts from madder roots (*Rubia tinctorum*), a natural dye plant for textiles known since the Iron Age [9].* R. tinctorum* contains a mixture of various anthraquinone derivatives with the most prominent colourant being alizarin (1,2-dihydroxyanthraquinone) that can appear in madder as some percent of the plants dry matter and is the main source of the red colour of these roots [10–12]. Alizarin itself has no strong affinity to the protein structure of keratin based fibres like wool and therefore requires the presence of a metal cation as mordant to form a stable coordination complex that enables stable incorporation within the fibre matrix [13–15]. The most common mordants for alizarin in Anatolian dyeing procedures are potassium alum (KAl(SO_4_)_2_) and iron(II) sulfate (FeSO_4_) [1, 3]. It is assumed that alizarin forms 2 : 1 complexes with trivalent (and some bivalent) metal cations via its adjacent keto- and hydroxyl-groups [13]. While the aluminium complex exhibits an intense red hue that is close to the shade of pure alizarin, the iron complex appears brown to black and can be transferred to violet by applying cinders of wood [1]. Since the fermented wool fibres are exposed to mordant and madder extract simultaneously within the dyeing bath, it can be concluded that the enrichment of the respective metals within the keratin matrix provides a direct detection of mordant-dye-complex distribution and, therefore, enables identification of fermentation of the wool fibre prior to dyeing.

A suitable method for quantitative element-specific analyses with high spatial resolution is X-ray fluorescence microscopy (*μ*-XRF) [16–18]. The method has proven its value for elemental mapping in cultural heritage studies, regarding, for example, paintings [19–22], inks [23, 24], and solid objects [25, 26], and also enables a detailed investigation of oxidation states by performing X-ray absorption near edge spectroscopy (XANES) [27–31]. Although many biological tissues are prone to decomposition upon X-ray radiation [32–34], sheep wool and other keratin based biofibres are relatively stable to significant X-ray doses and are not expected to be structurally destroyed during standard *μ*-XRF mapping [35].

We have investigated the quantitative mordant distribution in several recently prepared sheep wool fibres dyed with madder root extracts and either potassium alum or iron(II) sulfate mordant by means of *μ*-XRF and compared them to control specimens. The results of these experiments are employed to prove the fermentation procedure for an ancient wool specimen from an 18th century Anatolian carpet. Furthermore, we present a spectroscopic analysis of the oxidation states of the metal ions within the mordant-dye-complex and conclude influences on the shade of the resulting fibre colour.

## 2. Experimental and Data Analysis

The process of dyeing for the recently prepared wool fibres is described in detail by Bieber [3]. The basis of the fermentation process is a suspension of sourdough and wheat bran. The latter fosters selective growth of* G. candidum* yeast due to a high content of pentosan. The* G. candidum* culture regulates the pH to 4.4 and hinders the growth of putrefactive bacteria. Within about one week of fermentation* G. candidum* decomposes lipids inside the cuticle layers of the wool enhancing permeability within the subsequent dyeing process. After fermentation madder roots and mordant are added to the suspension at ambient conditions. Red fibres were achieved within a 20% solution of KAl(SO_4_)_2_, while a 10% solution of FeSO_4_ yielded almost black yarn.

The ancient specimen was extracted from red yarn of an 18th century Anatolian carpet. It was supposed that this wool had been dyed with madder and aluminium mordant after previous fermentation.

The various specimens were embedded in an epoxy resin derived from a 1 : 1 mixture of 4,4′-methylenebis(2-methylcyclohexylamine) and trimethylolpropane triglycidyl ether. After drying the resin at 60°C for 10 h, the sample blocks were partially microtomed to obtain a flat cross-section surface along the diameter of several isolated wool fibres.

*μ*-XRF studies were performed at the PHOENIX beamline at the Swiss Light Source in Villigen, Switzerland. The X-ray beam was focused to 3 × 5 *μ*m^2^. The excitation energy for fluorescence mapping was 3.0 keV for Al* K*-edge mapping and 7.2 keV for Fe* K*-edge mapping. The samples were mounted under 45° degree relative to the incoming beam to allow penetration of X-rays into the sample and an exit of the elemental fluorescence signals from the specimen (penetration depth: 45 *μ*m at 3.0 keV, 597 *μ*m at 7.2 keV). Images were taken by scanning the sample relative to the fixed position of the microbeam. The energy dispersive X-ray fluorescence spectra were recorded for each position using a single-element solid state detector (Ketek) with 160 eV energy resolution. By optimizing the fluorescence yield a suitable signal-to-noise ratio could be achieved, although the metal content within the samples was close to detection limit.

The standards for quantitative analysis were a 2 nm Al_2_O_3_ and a Fe_2_O_3_ film prepared by atomic layer deposition on a silicon wafer. The analytical grade powder Fe(II) and Fe(III) standards used for the Fe oxidation state were measured in total electron yield in order to avoid overabsorption artefacts of the spectra.

Fluorescence maps have been quantitatively fitted and analysed with PyMca [36], while XANES spectra have been analysed with Athena [37]. The attenuation of the excitation and fluorescence photons within the hair fibres was calculated for each excitation energy based on literature values [38]. With this method we could calculate the energy dependent probing volume and correlate the fluorescence signals to the quantitative metal contents. For determining the Fe oxidation state by scanning the incident energy, the measured Fe K-fluorescence line was first normalised against incident intensity (I_0_) and then normalised to a postedge value of 1.

## 3. Results and Discussion

**Figure 2:**
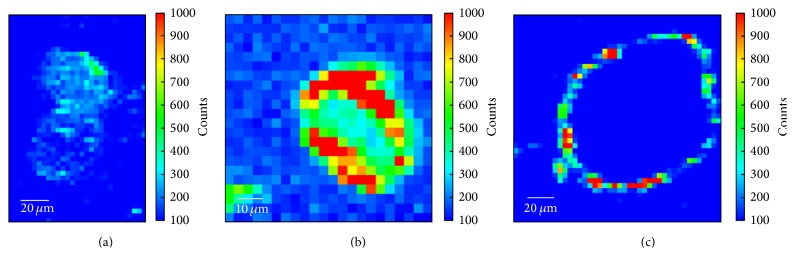
Fitted X-ray fluorescence maps at the Al* K*-edge (3.0 keV excitation energy, 2.8 s acquisition per pixel). (a) Recently dyed with madder roots and KAl(SO_4_)_2_. (b) 18th century carpet. (c) Blank sample without KAl(SO_4_)_2_ treatment, but Al contaminations at the surface.

**Figure 3:**
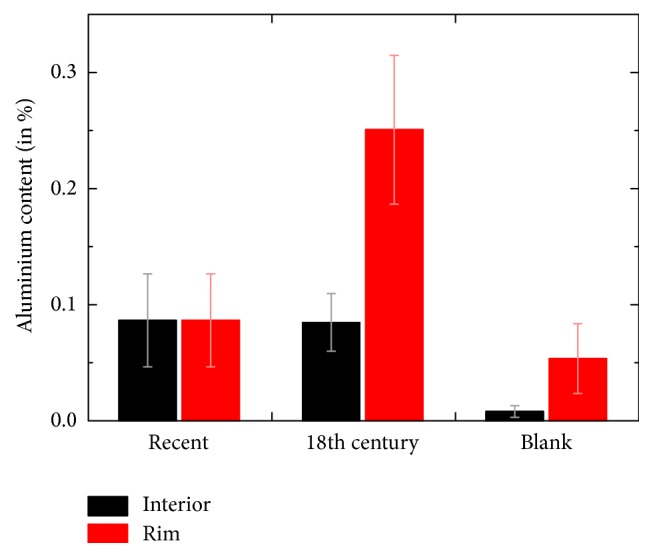
Calibrated Al content for the three different specimen types depicted in Figure 1 (average values for several single fibres).

Exemplary Al* K*-edge maps are depicted in Figure 2. Individual hair fibres appear as oval shaped objects with diameters in the range of 50–80 *μ*m. Although the sheep hair cortex is clearly separated into an ortho- and paracortex (cf.  Figure 1(d)) [8, 35, 39], we do not detect influences of these two regions within the elemental maps.  Figure 2(a) shows two fibres from the recently dyed red specimen with Al mordant. Although the metal content is quite low in the lower fibre, both have a significant fluorescence signal in the central region and the mordant distribution is relatively homogeneous over the whole fibre diameter (clearly visible at least for the upper fibre). In Figure 3 we see that the average Al content within this sample type is 0.09%. Furthermore, there is no apparent difference between the average metal content in the inner hair cortex and the cuticle for this batch. In the case of the blank sample (Figure 2(c) and Figure 3, right column) the Al content in the cortex is below the quantitative detection limit. However, we find Al contaminations on the rim of the cuticle for almost each fibre, which we attribute to unfavourable storage conditions. We can conclude that the relatively high Al content within the cortex of the first specimen is a characteristic feature of wool that has been fermented prior to dyeing. Note that it is important to use blank samples from the same breed or at least the same geographical region as the specimens, since metal contents in mammalian hair are strongly influenced by the environmental conditions [40, 41].

When we compare these results with the specimen from the 18th century carpet (Figure 2(b) and Figure 3, middle column), we find an almost identical Al content in the fibre cortex with respect to the recently dyed batch. However, the cuticle Al content within the rim of the fibres is significantly enhanced (up to 0.26%). A comparison with the respective S map shows clearly that this Al is present within the fibre and not at the surface as in Figure 2(c). Since the exact specifications of ancient dyeing suspensions are not known, we can speculate that the mordant concentration might have been higher compared to the preparation of the recent specimen. Despite this deviation, we still find a complete penetration of the wool fibres of this batch with mordant. This result clearly indicates that the 18th century wool has also been fermented and subsequently dyed with an Al mordant. In this case we are able to conclude on the manufacturing process of the yarn, although the cuticle scales are already scrubbed off the fibres and cannot be analysed anymore.

**Figure 4:**
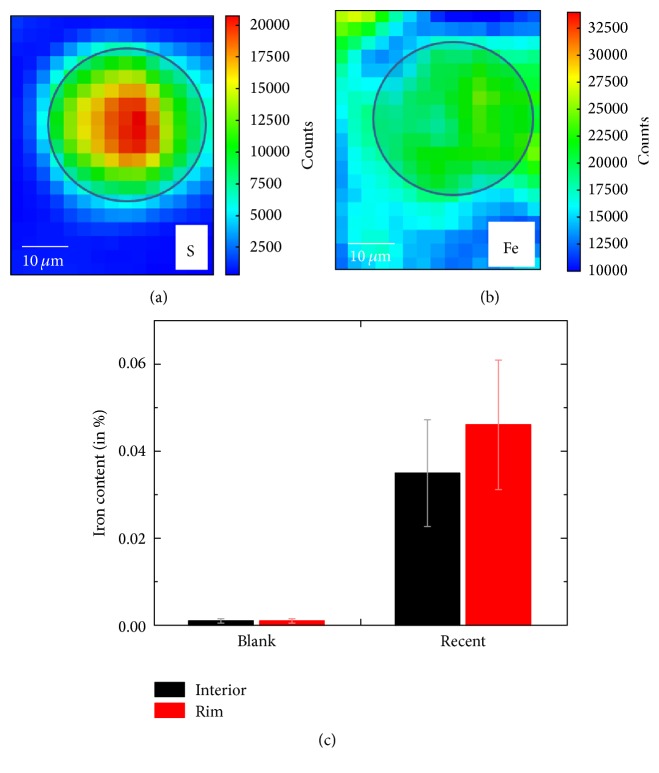
Wool fibres recently dyed with madder roots and FeSO_4_. (a) and (b) Fitted X-ray fluorescence maps of an exemplary fibre at the S and Fe* K*-edges (7.2 keV excitation energy, 1.6 s acquisition per pixel). (c) Calibrated Fe content for the Fe mordant containing specimen and a blank sample (average values for several single fibres).

Figures 4(a) and 4(b) illustrate *μ*-XRF maps of a recently manufactured wool fibre dyed with madder roots and FeSO_4_, resulting in black colouring. Due to the higher penetration depth of X-rays at the excitation energy required for this analysis (7.2 keV), not only the fibre cross-sections as revealed by the microtome sectioning, but also artefacts from the progression of the fibres further inside the embedding epoxy are visible. Therefore, the positions of suitable fibre cross-sections have been determined in the respective S map, while the corresponding Fe map was used for analysis. To achieve correct quantitative data, we selected only fibres that appeared almost orthogonal towards the surface for detailed analysis. Similar to the recent Al containing specimen (Figure 2(a)) we detect a relatively homogeneous distribution of Fe mordant along the fibre diameter. This is again caused by the influence of wool fermentation as discussed above. The average iron content within this batch was 0.04% with a small increase for the rim of the fibres, while the Fe content in the corresponding blank sample was below the detection limit (Figure 4(c)).

**Figure 5:**
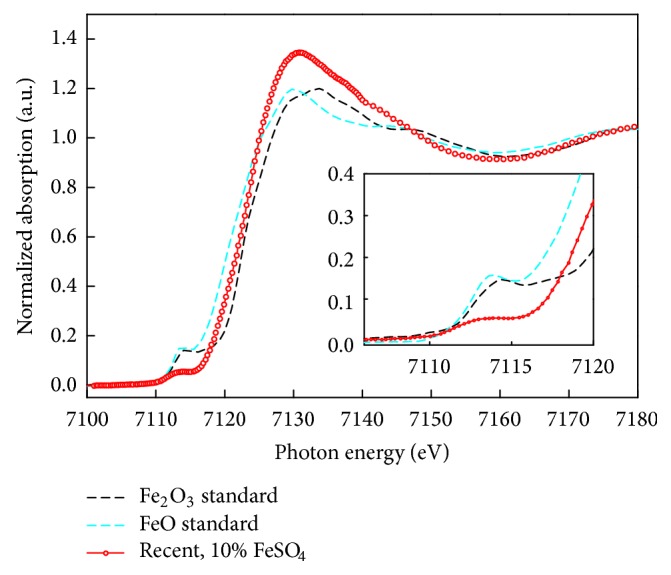
Fe* K*-edge XANES spectra in fluorescence yield of wool fibre dyed with FeSO_4_ mordant (red) compared to Fe_2_O_3_ (black) and FeO (light blue) standards measured in total electron yield. The* K*-edge peak for the specimen is shifted towards higher photon energy compared to the FeO standard, suggesting an increase in Fe^3+^ content.

In addition to elemental mapping, we recorded* K*-edge XANES spectra to detect the oxidation states of the investigated metal mordants within the wool specimens. In the case of KAl(SO_4_)_2_ mordant, we found Al^3+^ (not shown), which is usually the only stable not neutral oxidation state of Al in ambient conditions and corroborates the structure of the coordination complex of alizarin and Al proposed in literature [13]. When FeSO_4_ is used as mordant, a significant colour change from red to black is achieved (a similar behaviour is also found for yellow dyes, e.g., luteolin from* Reseda luteola*, which turns into brown with Fe mordant) [1, 3]. This behaviour is a hint on a stronger electronic interaction of the metal ion and the conjugated *π*-system of the dye within the resulting coordination complex. During the bating process the wool fibres are exposed to Fe in oxidation state +II. The standard method to differentiate between oxidation states +II and +III in Fe* K*-edge XANES is the relative position of the main absorption edge and the shape of the 1s→3d preedge peak [28, 42, 43]. This method provides quantitative information if the coordination geometry of the investigated metal complex is known and clearly defined [44]. In the present case both conditions are not fulfilled, but a qualitative analysis of the oxidation state within the mordant-dye-complex based on the centroid of the 1s→3d signal is possible [45]. We recorded XANES spectra from pure FeO(Fe (II)) and Fe_2_O_3_(Fe(III)) powders and for wool fibres recently dyed with FeSO_4_ (cf.  Figure 5). While the XANES spectra of pure Fe(II) and Fe(III) standards show a difference in edge positions of around 2.4 eV, the wool specimen spectrum is shifted to ~1.0 eV higher photon energy compared to the Fe(II) and the spectrum consists of mixed Fe(II) and Fe(III) portions. The change in shape of the preedge peak and main absorption line observed for the wool sample is due to a disordered first oxygen shell surrounding the Fe atoms compared to a perfect crystalline order present in the Fe standards which give rise to well defined absorption bands. These results, in particular the intermediate shift of the 1s→3d centroid (1.0 versus 2.4 eV), suggest the presence of a significant portion of Fe^3+^ within the coordination complex of alizarin, while a contribution from Fe^2+^ is still obvious. We conclude a partial charge transfer from the metal ion to the conjugated *π*-system of the dye that contributes to the significant colour change in mordant-dye-complexes of Fe.

## 4. Conclusions and Outlook

By means of *μ*-XRF elemental mapping we showed that the increased cuticle permeability of fermented sheep wool leads to a deeper and more homogeneous penetration of the wool fibres with the colouring dye-mordant complex, resulting in outstanding colour brilliance of the respective Anatolian textiles. Furthermore, it is possible with this technique to prove the fermentation procedure for ancient specimens that have lost the cuticle layers of the individual fibres over time. This is an important finding for the analysis of woollen cultural heritage. So far, the fermentation technique is only known to be used in a confined area in Anatolia within modern age and it is not clear since when it has been applied. The investigation of selected benchmark specimens with *μ*-XRF could be employed to address these open issues and enlighten the history of traditional preindustrial dyeing techniques. Furthermore, this investigation could trigger new developments in industrial colouring, since the persisting colour brilliance of fermented Anatolian wool is still outstanding.

The XANES investigations hint on a partial charge transfer from the metal ion to the conjugated *π*-system in mordant-dye-complexes of Fe. This finding might explain the significant change in colour when Fe mordants are used compared to Al. However, these results have to be corroborated by further studies on a broader range on mordant-dye-complex systems. Furthermore, the metal contents of the investigated specimens have been comparably low and higher concentrations would strongly increase the quality of the XANES spectra that are close to the detection limits so far.
